# A Case of Tetanus, in Which Large Doses of Opium Were Successfully Used

**Published:** 1823-12

**Authors:** G. Harvey

**Affiliations:** Surgeon, Castle Hedingham, Essex.


ART. II.—*A Case of Tetanus, in which large Doses of Opium were successfully used.* By G. HARVEY, Esq. Surgeon, Castle Hedingham, Essex. [Communicated through Dr. JAMES JOHNSON.]

SEPTEMBER 8th, 1823, John Johnson, a wheelwright, aged about twenty, nearly cut off the extremity of the middle finger of the left hand, with an axe. The wound extended about half way across the nail, near its root; the bone was completely divided, and the artery bled so profusely, that I was obliged to secure it by ligature. The wound was closed by slips of adhesive plaster, and for some days union by the first intention appeared probable. The ligature came away with the dressings on the eighth or ninth day. About this time he was dressed, daily, with ungt. resinæ, the wound beginning to slough and look unhealthy: however, he suffered little or no pain. On the 24th, my assistant, after dressing him, informed me that he complained of stiffness in his jaw, and a little soreness of throat, for which he had given him a calomel bolus and an aperient draught, supposing the symptoms were caused by cold; but the patient himself imagined they were occasioned by his having eaten a large quantity of nuts. Judging from this account that he was affected with tetanus, I called on him the following morning, and found his jaw so nearly closed that I could not introduce the point of my fore-finger further than about half-way up its nail. The muscles of the neck, particularly the mastoidei, felt rigid ; he complained of great stiffness about the neck and jaw, but had no difficulty of swallowing: the wound caused little or no pain. I immediately gave him sixty drops of tinct. opii, and ordered the same dose to be repeated at bed-time, and the finger to be wrapped in a bread-and-water poultice. The opening medicine given yesterday operated several times.

26th.—No material alteration; says he slept well, but that his neck and shoulders are very stiff. Desired him to remain in bed, in order that perspiration may be promoted.

℞. Pulv. Opii, gr. vj.; Camph. ℈j.; Calomel, gr. iij.; Conf. Opii, q. s. div. in pilulæ xij. Capt. j. 2da. q. h. cum cochl. ij. mist. sequentis. —℞. Sp. Æth. comp. ʒj.; T. Lav. comp. ʒss.; Mist. Camphoræ, ℥xj. M.

27th.—Something better to-day. I can now introduce the point of my finger into his mouth, nearly up to the first joint; stiffness extending between the shoulders, but he can readily bring his chin down to the sternum, and move his head in all directions. No difficulty of swallowing, or spasmodic paroxysms, but a constant aching pain; pulse natural, tongue furred, thirst moderate. Repetantur medicamina. Ordered to take wine and nourishing broths, but to remain in bed.

28th.—Much the same; no stool since the 25th. Capt. statim Infus. Sennæ, ℥ij. alvô solutâ. Rep. pilulæ et mistura.

29th.—Bowels constipated: in other respects as yesterday. Rep. Infus. Sennæ, statim.

℞. Aloes—Camphoræ āā ʒss. M. Div. in pilulas No. xij. capt. ij. 2d q. h. donec alvus respond.

Oct. 2d.—Bowels have been very freely relieved. He can now open his mouth sufficient to admit my finger with tolerable facility; but the muscles of the neck and back are so powerfully contracted, that he can neither move his head, which is strongly drawn backward, nor bend his body; complains of great pain in his shoulders and hips. A large blister to be applied between the shoulders, and another to the lumbar region.

℞. Pulv. Opii, Camphoræ, Pulv. Ipecacuanhæ, āā gr. xxiv.; Extr. Hyosciami, q. s. div. in pilulæ xij. Capt. j. 2da q. h. c. cochl. iij. Mist. sequentis.—℞. Sp. Æth. comp. Sp. Ammon. comp. T. Lavend. c. āā ʒss.; Mist. Camph. ℥xss. M.

3d.—Something better to-day. Removed the slough and carious bone; wound granulating, and gives very little pain; says he sleeps well; has no head-ache, thirst, or tendency to fever; moves his arms and legs with ease, but cannot bend his body or raise his thighs. No stool since the 1st. Capt. Calom. gr. iij. h. s. Infus. Sennæ, ℥ij. primo mane. The other medicines to be omitted till the bowels have been evacuated.

5th.—No material alteration; the bowels still costive. A common glyster to be injected, and repeated in the evening, if necessary.

7th.—Much the same; lies like a log in the bed, having no power to move his head or trunk of the body. Two enemata were thrown up, but with great difficulty, in consequence of the strong muscular action, and caused so much pain that he begs they may not be repeated. Capt. statim Ol. Ricini, ℥j. et rep. post horas tres si opus sit.

℞. Ext. Hyosciami, T. Opii, āā ʒj.; Spt. Æth. comp. ℥ss.; Aq. Menthæ, ℥vj. M. Capt. cyathum, (a wine-glassful,) 3 q. h. incipiens post operationem Ol. Ricini.

8th.—Both doses of castor oil were taken, and produced several evacuations. Rigidity still continues unabated. Repetatur mistura, addend T. Opii, ʒss.

9th.—No abatement of rigidity, except that he opens his mouth with rather more freedom. Dislikes the mixture, several doses of which have been immediately rejected; very little sleep. Capt. Opii nud. gr. iv. statim.

℞. Tinc. Opii, ʒiij.; Tinc. Lavend. c. ʒij.; Aq. Pulegii, ℥viij. M. Capt. ut antea.

10th.—No sickness since yesterday; rigidity much the same, but less pain.

℞. Pulv. Opii, Camphoræ, āā ℈j. M. divide in pilulas viij. Capt. ij. c. sing. dos misturæ.

11th.—Much better in every respect. Sleeps well, but not more than patients usually do after a full dose of opium. Rep. pilulæ et mistura. The castor-oil to be repeated, if necessary.

13th.—Has omitted several doses of his mixture and pills, having slept. Castor-oil repeated yesterday, with good effect. Can raise himself a little, but not sufficiently to sit up. Rept. mistura et pilulæ.

15th.—Much better; managed to sit up, with some difficulty.

His mixture and pills were continued till the 20th, when I found him so far recovered as to be able to walk across the room. The last two days he has slept almost continually. Has been sick several times this morning, and perspires profusely; very little pain or rigidity; opens his mouth nearly to its full extent. Conceiving now the further use of opium to be unnecessary, I discontinued it altogether, and ordered the following mixture, and a continuation of his wine and nourishing diet.

℞. Spirit. Ammoniæ c. ʒij.; Mist. Camph. ℥iij.; Infus. Gentian. comp. ℥v. M. Capt. cyathum ter in die.

He continued this plan till the 28th, when, being perfectly recovered, I desired him to discontinue all medicines; dressed his finger (which had hitherto been poulticed,) with unguent. resinæ; and recommended him to use daily exercise in the open air.

